# Effect of Nano Copper on the Densification of Spark Plasma Sintered W–Cu Composites

**DOI:** 10.3390/nano11020413

**Published:** 2021-02-05

**Authors:** Vadde Madhur, Muthe Srikanth, A. Raja Annamalai, A. Muthuchamy, Dinesh K. Agrawal, Chun-Ping Jen

**Affiliations:** 1 Centre for Innovative Manufacturing Research, VIT, Vellore, Tamil Nadu 632 014, India; madhur.vadde@gmail.com (V.M.); muthe.srikanth@vit.ac.in (M.S.); raja.annamalai@vit.ac.in (A.R.A.); 2 Department of Metallurgical and Materials Engineering, National Institute of Technology Tiruchirappalli, Tamil Nadu 620015, India; muthuchamy@nitt.edu; 3 Materials Research Institute, The Pennsylvania State University, University Park, PA 16802, USA; dxa4@psu.edu; 4 Department of Mechanical Engineering and Advanced Institute of Manufacturing for High-Tech Innovations, National Chung Cheng University, Chia-Yi 62102, Taiwan

**Keywords:** tungsten-*(nano)* copper composites, solid-state sintering, spark plasma sintering, microstructure, mechanical properties

## Abstract

In the present work, nano Cu (0, 5, 10, 15, 20, 25 wt.%) was added to W, and W–Cu composites were fabricated using the spark plasma sintering (S.P.S.) technique. The densification, microstructural evolution, tensile strength, micro-hardness, and electrical conductivity of the W–Cu composite samples were evaluated. It was observed that increasing the copper content resulted in increasing the relative sintered density, with the highest being 82.26% in the W75% + Cu25% composite. The XRD phase analysis indicated that there was no evidence of intermetallic phases. The highest ultimate (tensile) strength, micro-hardness, and electrical conductivity obtained was 415 MPa, 341.44 HV_0.1_, and 28.2% IACS, respectively, for a sample containing 25 wt.% nano-copper. Fractography of the tensile tested samples revealed a mixed-mode of fracture. As anticipated, increasing the nano-copper content in the samples resulted in increased electrical conductivity.

## 1. Introduction

Refractory metal tungsten (W) has excellent thermal creep resistance, high electrical and thermal conductivity, and the lowest vapor pressure among all metals. On the other hand, copper (Cu) has superior electrical and thermal conductivity and has good corrosion resistance. When copper is added to tungsten, a binary system in which the two metals are mutually insoluble [1] is formed. This insolubility is due to incompatibility in their crystal structures. W is body-centered cubic (BCC), and Cu is face-centered cubic (FCC), and the two have a large difference in their densities, melting points, and electronegativity. These pseudo alloys have exceptional properties such as high strength, good wear resistance, high arc erosion resistance, low thermal expansion coefficient as well as high thermal and electrical conductivity, thereby making them applicable for high-performance electrical contacts, heat sinks, plasma-facing materials in nuclear fusion reactors, electrodes for Electrical Discharge Machining (EDM), radiation shielding materials, and microwave communication systems [2,3,4,5,6,7,8,9,10,11,12,13]. Due to the high processing temperature of tungsten and mutual insolubility, it is difficult to process these pseudo-alloys using conventional methods, hence, techniques such as liquid infiltration and liquid phase sintering are employed to sinter them [14,15,16]. However, it is not easy to obtain full densification even through these processes.

Therefore, to ensure full densification, either high temperatures, high compaction pressure, longer holding time, or the addition of other transitions elements (namely Fe, Ni, or Co) are required. High temperatures or long holding time results in a non-homogeneous microstructure due to the copper leaching out. High compaction pressure limits the particle rearrangement due to increased particle–particle contact. The addition of Ni, Fe, and Co results in low electrical and thermal properties. From various experimental investigations, it has been found that the starting particle size and uniformity of the tungsten and copper powders significantly affect the magnitude of the sintering temperature and sinterability, and improve the final properties of the W–Cu composites [17,18,19,20].

Similarly, temperature and powder particle size are two main factors that determine sinterability. High-energy mechanical milling of tungsten and copper powders to produce fine or ultra-fine particles resulted in high relative density and lower sintering temperatures [3,20]. Processing techniques such as high-pressure sintering, microwave sintering, dynamic consolidation, and low-temperature infiltration in supergravity fields have resulted in higher density, improved mechanical properties, and a significant reduction in processing time [2,3,15,21,22,23]. As the copper content increased in the W–Cu composite, the electrical conductivity increased, as expected [21]. However, despite the higher copper content, the electrical conductivity reduced as a result of higher sintering temperature [20]. To overcome the drawbacks of liquid phase sintering, conventional sintering, and reduce the processing time and temperature, SPS. was employed in the current work to fabricate W–Cu composites at 900 °C. As the starting particle size affects the sintering temperature, as-received nano-copper powder was used in this study.

## 2. Experimental

### 2.1. Materials

**Figure 1:**
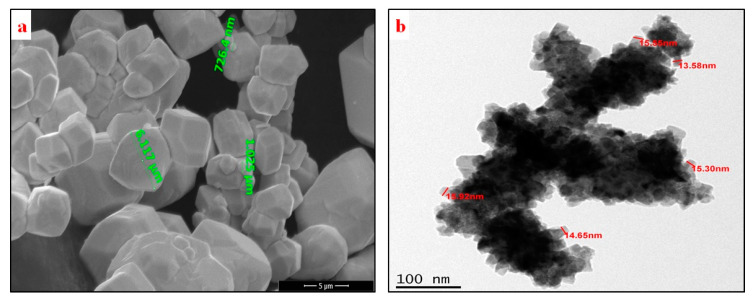
Powder morphology of as-received (**a**) tungsten powder and (**b**) nano-copper powder.

Nano copper powder (Sigma-Aldrich, Bengaluru, India) with a particle size less than 100 nm and 99.99% purity and tungsten powder (Sigma-Aldrich, Bengaluru, India) with a particle size of ~12 µm were used as an as-received form. Figure 1a shows the powder morphology and size of the as-received tungsten powder as observed with the field emission scanning electron microscope, and Figure 1b shows nano-copper powder morphology and particle size as observed with a transmission electron microscope. The W–Cu (0, 5, 10, 15, 20, 25 wt.%) mixtures were prepared by weighing the required amounts of powders, followed by manual mixing in an agate mortar for 10 min and mixing in a planetary ball mill for 15 min in the dry state at 200 rpm without balls. The mixture was transferred into the SPS machine for sintering. The surface of the punch and die was separated from the powder mixture using a thin graphite sheet.

### 2.2. Spark Plasma Sintering (SPS)

SPS can be used to produce sintered samples rapidly in a single step using a combination of pressure, temperature, and electric field. In contrast to conventional sintering and other novel sintering techniques, SPS can significantly reduce the overall processing time through its high heating rates (about 100 °C per minute). Depending on the graphite die and punch geometry, very high heating rates such as 1000 °C per minute can also be attained. This is possible because of the electrical conductivity of the tool materials used in the SPS process. The low voltages applied across the set-up produce high currents, resulting in effective joule heating. Simultaneously, a uniaxial load is applied to the powder mixture to enhance the densification [24,25]. The sintering temperature of 900 °C in a vacuum with a heating rate of 100 °C per minute at a uniaxial pressure of 50 MPa in a graphite die of 30 mm diameter (model: Dr. Sinter 21050, supplier: Suji Electronic Industrial Co, LTD, Japan) was used. A voltage of 30 V and a current of 600 A was applied to process the compacts. A thin graphite sheet was used to separate the powder mixture from the surface of the die. Subsequently, after reaching the desired temperature of 900 °C, the samples were soaked for 120 s. After that, the furnace was turned off, and the compacts were left in the furnace to cool to room temperature.

### 2.3. Characterization of Sintered Samples

The density of the sintered samples was calculated using the Archimedes principle with three iterations for a sample. The samples were initially grounded using Emery sheets (grit size 220, 400, 600, 800, 1000, 1200, 1500, and 2000, respectively) and then polished to scratch-free surface using polishing machine (supplier: Chennai Metco Pvt. Ltd, Chennai, India) in the alumina media. According to the ASTM E407 standard [26], the polished samples were etched using Murakami’s reagent containing 100 mL of water, 10 g of potassium hydroxide (supplier: Finox Pellets Industries, Gujarat, India), and 10 g of potassium ferricyanide (supplier: Hemadri Chemicals, Mumbai, India) to obtain the optical micrographs and thereby to determine the grain size of the samples. The line intercepts method was used on optical micrographs at 500x magnification. The samples’ SEM images were obtained in both secondary electron mode and back-scattered electron mode (model: E.V.O. 18 Research, supplier: Zeiss, Oberkochen, Germany). X-ray powder diffractometry was used to analyze the sintered samples’ phase composition (model: X’Pert^3^ Powder, supplier: Malvern PANalytical, Malvern, UK) with a scan speed of 10 degrees per minute. Micro-tensile test specimens were machined from the sintered samples using wire-cut EDM and were tested with a crosshead speed of 1 mm/min (model: 8801, supplier: Instron, Norwood, MA, USA). The Vickers’s micro-hardness of the samples was measured with a load of 0.1 kgf and with a dwell time of 10 s according to ASTM E92 [27] (model: MMT-X, supplier: Matsuzawa Co., Ltd, Japan). On each sample, twenty indentations were made randomly across the cross-section to obtain a mean value. The samples’ electrical conductivity was measured according to ASTM E1004 [28] using the eddy current probe set up (Supplier: Technofour, India).

## 3. Results and Discussion

### 3.1. Density

The increase in *nano*-copper content in the compacts eventually led to increased relative density, with the highest being 82.26% for the C6 (refer to Table 1) compact. It is known that the increase in surface area increases the flow of powder particles. A combination of pressure and electric current enhanced the sinterability, even at a lower sintering temperature in the present work. Furthermore, the use of *nano*-copper powder facilitated the sinterability. The increase in relative sintered density of the compacts can be attributed to the flow of semi-solid *nano*-copper into the pores between the tungsten–tungsten grains. There was no substantial increase in the relative densities between the C5 and C6 compacts, which can be the effect of agglomeration of the particles. However, the relative density results of the compacts showed that the final stage of sintering, which results in pore shrinkage, was not entirely accomplished.

### 3.2. X-ray Diffraction (XRD)

**Figure 2:**
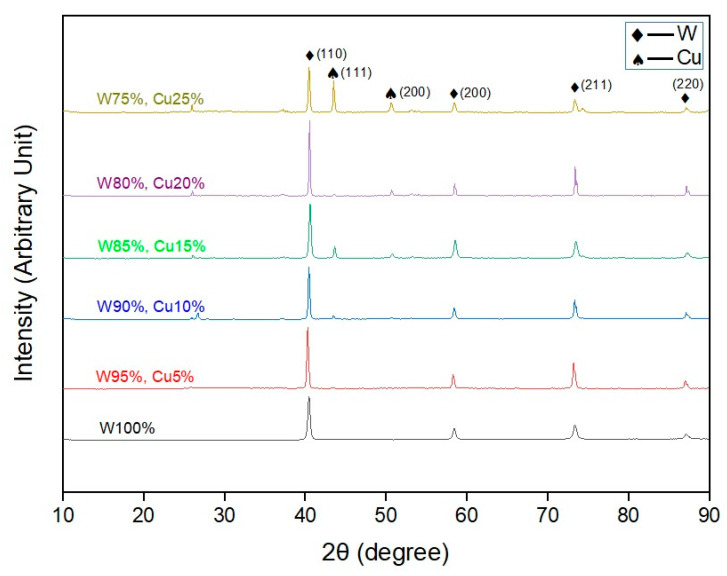
X-ray diffraction (XRD) profile of the sintered samples.

XRD patterns of the sintered samples are shown in Figure 2. It was observed that the position and intensity of the diffraction peaks matched the reference plots [29], indicating the presence of only W and Cu phases. No oxide phase was found in the XRD patterns, implying the success of sintering in vacuum conditions. From the plot, it is evident that copper peaks were enhanced, which indicate good (semi-solid) flow of the nano-copper to fill the pores and thus obtain good densification.

### 3.3. Microstructure

**Figure 3:**
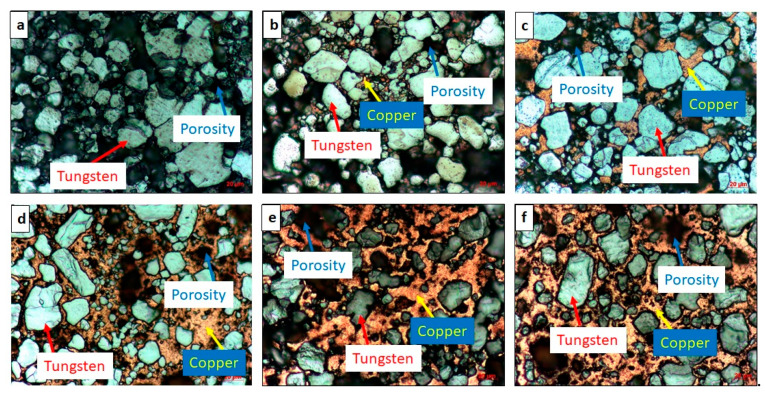
Optical micrographs of (**a**) W100%; (**b**) W95%, Cu5%; (**c**) W90%, Cu10%; (**d**) W85%, Cu15%; (**e**) W80%, Cu20%; (**f**) W75%, Cu25% compacts.

**Figure 4:**
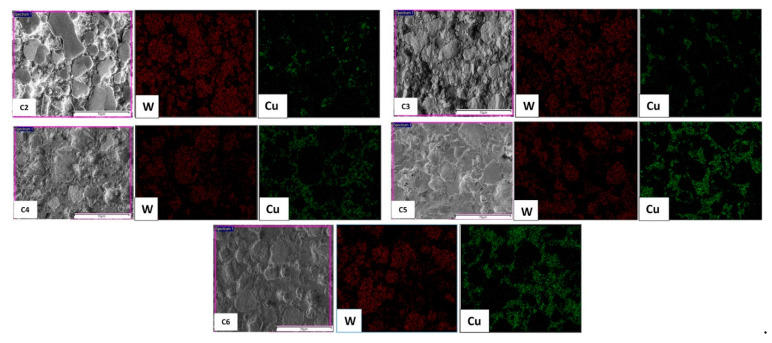
Elemental mapping of various compositions.

The optical micrographs of the sintered W–*(nano)*Cu compacts are shown in Figure 3. A two-phase microstructure with bright white tungsten grains embedded in a reddish-brown copper matrix was observed. In the compacts with lower *nano*-copper content (i.e., in C2, C3, and C4), an even distribution but a discontinuous network of copper phase was observed. In the C5 and C6 compacts, a more uniform distribution and continuous network of the matrix phase were observed, which also conformed to the elemental mapping shown in Figure 4. This might have contributed to the enhancement in the densification and reduction of porosity. Due to the low solubility of either element in one another, aggregation of *nano*-copper powder particles around the tungsten grains was observed. The compacts’ grain size is listed in Table 2; the base tungsten compact (C1) had an average grain size of (20 ± 2) µm, which was reduced to (15 ± 1) µm of the C6 compact. This reduction in the compacts’ grain size (C1 through C6) can be attributed to the increased *nano*-copper content, which inhibited the tungsten grain growth, resulting in relatively refined grains.

### 3.4. Mechanical Properties (Behavior)

**Figure 5:**
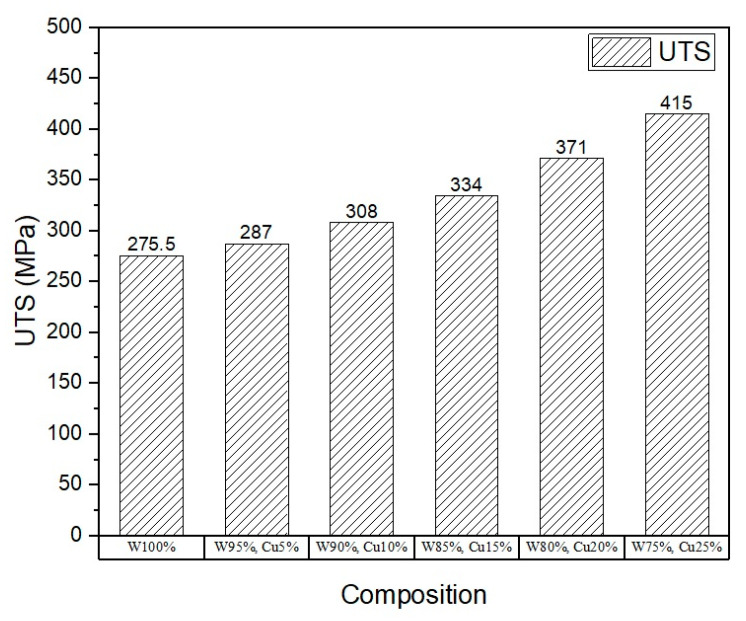
Plot of ultimate tensile strength.

**Figure 6:**
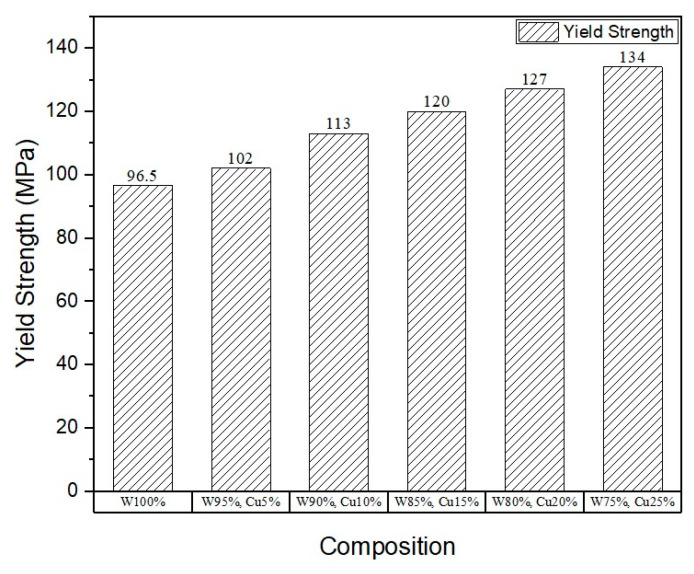
Plot of yield strength.

**Figure 7:**
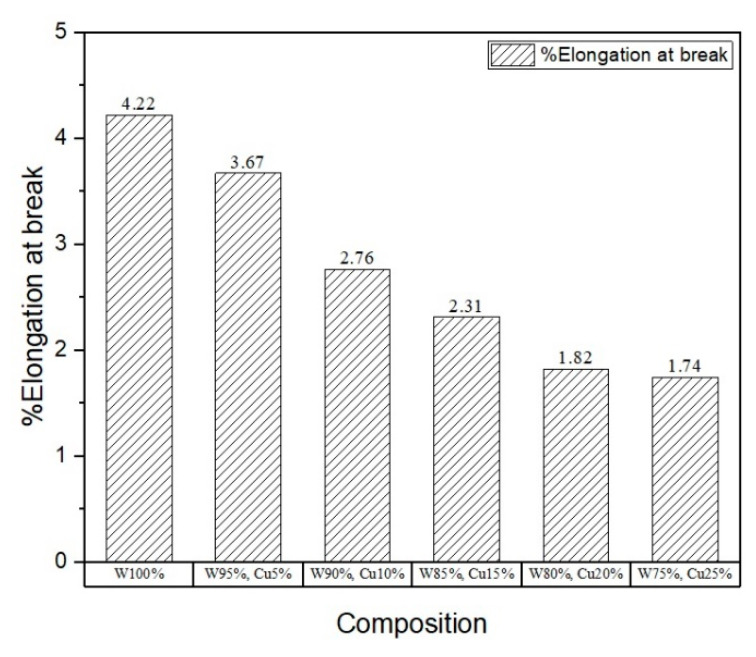
Plot of %elongation.

**Figure 8:**
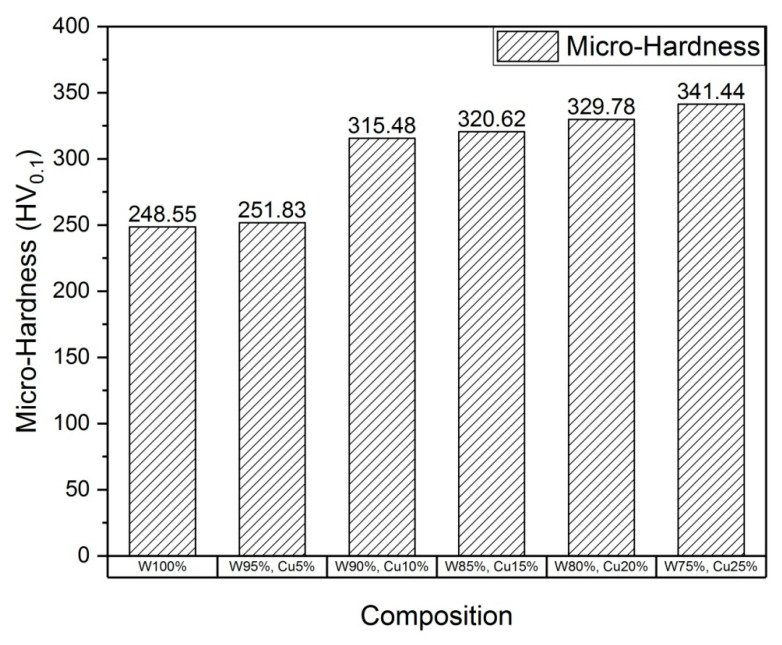
Plot of micro-hardness.

To assess the mechanical properties, micro-tensile and micro-hardness tests were carried out, and the results are shown in Figure 5, Figure 6, Figure 7 and Figure 8. The results show that the addition of *nano*-copper resulted in improved tensile strength and micro-hardness. Initially, there was no appreciable increase in strength or micro-hardness between samples C1 and C2. However, with the further increase in *nano-*copper content, significant improvement in the properties was observed, which was attributed to copper filling the pores and forming a network around the tungsten grains, thereby inhibiting grain growth and consequently increasing tensile strength (Figure 5) and reducing %elongation (Figure 7). From the Hall–Petch equation, it is well known that a decrease in grain size increases yield strength [30], which conforms to the obtained results, as shown in Figure 6. As a result, a nearly 50% increase in (tensile) strength and 37% increase in micro-hardness (Figure 8) were observed between samples C1 (W) and C6 (25% Cu). This increase can be attributed to both higher densification and grain refinement. The sample containing 25 wt.% *nano*-copper (C6) exhibited the highest tensile strength and micro-hardness values of 415 MPa and 341.44 HV_0.1_, respectively.

### 3.5. Fractography

**Figure 9:**
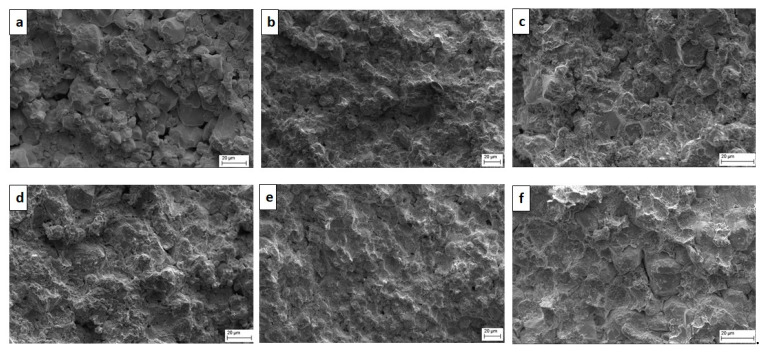
Scanning electron microscope (SEM) images of the fractured surface of samples tested for tensile strength. (**a**) W100%; (**b**) W95%, Cu5%; (**c**) W90%, Cu10%; (**d**) W85%, Cu15%; (**e**) W80%, Cu20%; (**f**) W75%, Cu25%.

**Figure 10:**
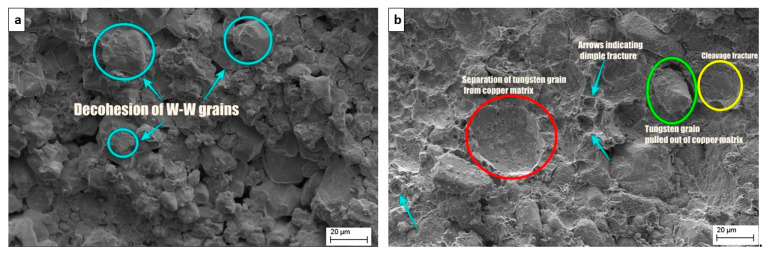
Image showing failure modes. (**a**) Base and (**b**) alloy with 25 wt.% nano-copper.

Figure 9 shows the SEM images of the fractured surface of the specimens tested for tensile strength. As expected, the brittle fracture was observed in pure tungsten; the failure started by separation of W–W grains and propagating along the grain boundaries. The addition of *nano*-copper to tungsten resulted in a mixed-mode fracture. Due to low wettability and low diffusion coefficient between tungsten and copper at 900 °C, sufficient inter diffusion did not occur [31] and as a result, tungsten grains are pulled out of the copper matrix. The inter-granular fracture was observed in the regions with low copper content. Cleavage fracture occurred at coarse tungsten particles. The tungsten grain regions, which were well surrounded by copper matrix, failed in trans-granular mode [32]. Decohesion of tungsten particles was observed, as shown in Figure 10a. Due to the different deformation rates between hard tungsten particles and the ductile copper matrix, voids are formed. Eventually, these voids grew and were linked up, resulting in dimple type fracture. In Figure 10b, different modes of failure can be observed including the separation of tungsten particles from the copper matrix, dimple fracture, cleavage fracture, and tungsten grain being pulled out of a copper matrix.

### 3.6. Electrical Conductivity

**Figure 11:**
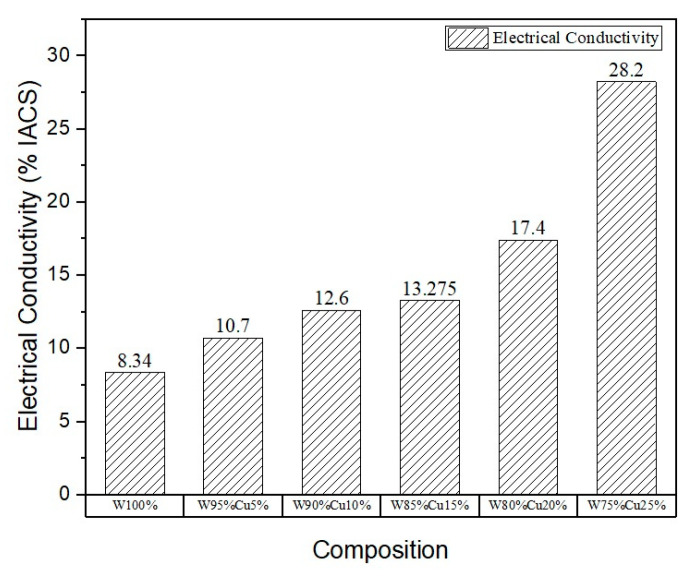
Electrical conductivity of the sintered samples.

The eddy current probe method was employed to measure the samples’ electrical conductivity, and the results are shown in Figure 11. The frequency of the alternating current is adjusted automatically by the device itself. As expected, increasing the *nano*-Cu content in samples resulted in increased electrical conductivity. However, the increase was not proportional to the increase in *nano*-Cu content. This can be attributed to the porosity of the samples, which restricts the flow of electrons. The highest electrical conductivity was found to be 28.2% IACS in the sample containing 25% *nano*-copper. The substantial increase in electrical conductivity can be attributed to the more uniform and continuous network of copper in the compact.

## 4. Conclusions

From the present study, it can be concluded that W–*(nano)*Cu composites can be fabricated with high density using the spark plasma sintering technique even at 900 °C. Properties such as relative density, tensile strength, micro-hardness, and electrical conductivity were found to increase with nano copper addition. No intermetallics were formed, as confirmed by XRD analysis. As the copper content increased, a more uniform and continuous network of the matrix phase was observed, reducing porosity. The microstructure observed that the third stage of sintering, in which shrinkage or closure of pores occurs, was not wholly achieved under the processing conditions used in the present study. However, an increase in uniaxial pressure or soaking time might overcome this and result in a further increase in relative density and other properties.

## Figures and Tables

**Table 1:** Composition of the samples, relative density, and their respective notations.

Composition (wt.%).	Relative Density (%)	Notation
W100%	69.76	C1
W95%Cu5%	71.89	C2
W90%Cu10%	77.34	C3
W85%Cu15%	79.91	C4
W80%Cu20%	81.43	C5
W75%Cu25%.	82.26	C6

**Table 2:** Grain size (of tungsten).

Composition.	Grain Size (µm)
W100%	20 ± 2
W95%, Cu5%	19 ± 2
W90%, Cu10%	18 ± 1
W85%, Cu15%	16 ± 1
W80%, Cu20%	16 ± 1
W75%, Cu25%	15 ± 1

## Data Availability

The data that support the findings of this study are available from the corresponding author upon reasonable request.
